# Diabetes Interactive Atlas

**DOI:** 10.5888/pcd11.130300

**Published:** 2014-02-06

**Authors:** Karen A. Kirtland, Nilka R. Burrows, Linda S. Geiss

**Affiliations:** Author Affiliations: Nilka R. Burrows, Linda S. Geiss, Division of Diabetes Translation, National Center for Chronic Disease Prevention and Health Promotion, Centers for Disease Control and Prevention, Atlanta, Georgia.

## Abstract

The Diabetes Interactive Atlas is a recently released Web-based collection of maps that allows users to view geographic patterns and examine trends in diabetes and its risk factors over time across the United States and within states. The atlas provides maps, tables, graphs, and motion charts that depict national, state, and county data. Large amounts of data can be viewed in various ways simultaneously. In this article, we describe the design and technical issues for developing the atlas and provide an overview of the atlas’ maps and graphs. The Diabetes Interactive Atlas improves visualization of geographic patterns, highlights observation of trends, and demonstrates the concomitant geographic and temporal growth of diabetes and obesity.

## Introduction

The National Diabetes Surveillance System (NDSS) provides county-level estimates of diabetes and selected risk factors for diabetes. Since 2008, when the first NDSS Internet application to display the geographic pattern of these county data was launched, the number of county health indicators, which include diabetes, obesity, and physical inactivity, has grown, and the number of years for which data are available has grown (1–3). Most recently, in 2012, county estimates for the incidence of diagnosed diabetes were added to the NDSS (4). As the number of indicators and years of county data increased, navigation of NDSS applications, which used a combination of static and dynamic maps, became difficult. Better visualization tools were needed to enhance understanding of geographic patterns, recognition of time trends, and identification of geographic and temporal growth of the relationship between diabetes and obesity. Thus, NDSS developed and released in 2012 the Diabetes Interactive Atlas (www.cdc.gov/diabetes/atlas/) to 1) improve visualization of the geographic pattern of diagnosed diabetes and its major risk factors at the national, state, and county levels; 2) illuminate trends in diabetes and its major risk factors at the national, state, and county levels; and 3) demonstrate the concomitant growth of diagnosed diabetes and obesity over time across the United States and within states.

## Design Considerations and Decisions

### Goal 1 — Improve visualization of geographic patterns

We designed the atlas to allow users to interactively manipulate the data and graphics at various geographic levels to enhance geovisualization, a process by which the brain recognizes geographic patterns and relationships (5). To map county data, we used a choropleth map, because it presents enumeration units as colors that symbolize ranges in data (6,7). Choropleth maps enable users to easily compare information across different geographies and recognize variability and patterns across a region (6,7). Two basic decisions in creating choropleth maps are color selection and data classification (6). For color selection, we chose sequential color schemes, where light to dark color sequences represent low to high values of prevalence or incidence. Sequential colors best represent order and are suited for low to high values on a numerical scale (eg, 0–6, 7–9, 10–18) or an ordinal scale (eg, below median, not above or below median, above median) (8). In our atlas, counties with the lowest prevalence of diabetes have the lightest color, and counties with the highest prevalence of diabetes the darkest color. We selected different sequential color schemes for each major indicator by using www.colorbrewer2.org, an online tool for selecting color schemes. The distinctive color scheme for each indicator provides a visual cue for users as they change indicators. When we chose colors for each indicator, we considered that potentially 1 in 12 users of the website may be color blind, and most blindness occurs in the red to green color schemes (9–11).

Data classification is the second major factor that needs to be considered when designing choropleth maps. Data classification is a method by which data are divided into intervals such as quantiles, natural breaks, or equal intervals. Two decisions to be made in data classification are the method of grouping the data into classes and the number of classes. In our atlas of county data, the default classes were determined by natural breaks for each indicator based on data from 2005, and the default number of classes was 5. We used natural breaks based on the Jenks method, which is designed to determine the best arrangement of values into different classes by reducing the variance within classes and maximizing the variance between classes (12). We used 2005 as the base year to create the natural breaks because this year was an early year of data classification, and the data could then be used to follow annual trends in subsequent years. Atlas users are given the ability to choose other data classification methods. For example, when viewing county data for a single year, the user can choose classes by quantiles, natural breaks, or equal intervals or can choose not to use classes but view data on a continuous scale. The user can select up to 10 data classes or breaks; however, for optimal viewing of data trends, no fewer than 4 and no more than 6 classes are recommended (13). Although maps and tables provide information powerfully, their analytical use is amplified when used collaboratively with other quantitative displays (14), allowing the user to view data from different perspectives simultaneously and to explore and detect geographic patterns. The Diabetes Interactive Atlas links maps to data tables, bar charts, line charts, and motion charts. All of these graphical components interact with the map to provide the user with various ways to view the data and explore geographic and temporal similarities and differences.

### Goal 2 — Illuminate trends

Because our first Internet application for county estimates displayed a separate map for each year of data, examination of trends was difficult. We addressed this issue in the new atlas by including time animation bars that play a succession of maps (or charts) over time — similar to an autoplay of a PowerPoint slideshow. Maps, tables, and charts are interrelated, so all change simultaneously when time animation is selected. The time animation bar also allows for switching between years of data and selection of a single year of data for further examination. Counties or states (depending on the map) that are highlighted will also be highlighted in the corresponding table or charts, allowing for easy tracking of these geographies over time. The motion charts also include a line chart that displays the median of the selected indicator by year. Users can select geographies (eg, several states) to compare their trend with the US median and determine how the trend might change.

### Goal 3 — Demonstrate the concomitant growth of diagnosed diabetes and obesity

One of the most powerful graphing displays for 2 indicators is motion charts (15). Motion charts, which are animated bubble plots, can display up to 5 parameters simultaneously: bubble size, bubble color, *x*-position, *y*-position, and time (15). Motion charts have enabled users to explore data interplays and investigate parameters in more detail than they can by using 2-dimensional static scatterplots (15). In our motion charts, each bubble represents a geographic unit (either a state or a county), bubble size represents the number of adults with the indicator (eg, the number of adults with diabetes), bubble color represents the data class, the *x*-position represents obesity prevalence, and the *y*-position represents prevalence or incidence of the indicator. The time animation bar plays the graph over time, showing how the 2 indicators have changed together. These motion charts were created primarily to demonstrate the concomitant growth of diabetes and obesity.

## Other Technical Considerations and Data Sources

Section 508 of the Rehabilitation Act mandates federal government agencies to develop accessible websites. An accessible website is designed in a way in which people with a disability can use it and obtain the same information that people without disabilities can obtain (16). Adobe Flash Player is required to view trends and motion charts in the atlas (17). According to US Department of Health and Human Services policy and standards for Web pages, Flash-based Web pages must make data, methods, and PowerPoint files available in a non-Flash environment (18). Excel data files used to create the atlas and PowerPoint files used to display maps were tagged and tested for 508 compliance (19). Headers, comments, notes, language, and titles were applied to all Excel files. We also created downloadable PDF versions of the Excel data as another way for users to access data. Titles, language, and alternative text used to describe images were applied to all PowerPoint files.

We use the Behavioral Risk Factor Surveillance System (BRFSS) to obtain estimates of prevalence and incidence of diagnosed diabetes and related risk factors at the state and county levels. State estimates are calculated directly from BRFSS, and county estimates are calculated according to Bayesian methods and small-area estimation using BRFSS and US census data (2,3). Estimates of diabetes prevalence are based on the number of respondents who answer yes to the question “Have you ever been told by a doctor that you have diabetes?” Sample size is sufficient to produce prevalence estimates by sex. To determine incidence of diabetes, respondents who answer yes in some states are asked additional questions from the optional diabetes module. Because the module is optional, some states have no data on incidence, and because of inherent sample size limitations, the variability in the number of new cases is too large to produce rates of incidence by sex (4). Estimates of obesity prevalence are derived from self-reported height and weight, where body mass index (weight [kg]/height [m^2^]) is 30.0 or greater. Estimates of physical inactivity are based on the number of responses of no to the question “During the past month, other than your regular job, did you participate in any physical activities or exercises such as running, calisthenics, golf, gardening, or walking for exercise?” We update the atlas as new BRFSS data become available.

We used InstantAtlas desktop version 6.5 software (Geowise Limited, Edinburgh, United Kingdom) to create the interactive Web pages. We used ArcGIS version 10.0 (ESRI, Redlands, California) to modify and project shapefiles for contextual and background maps used in the templates. The single-map template was used to create the Web pages for county data and county rankings. The bubble-plot template was used to create the Web pages for the maps and motion charts for all states together and for individual states.

## Content

### County data

**Figure 1:**
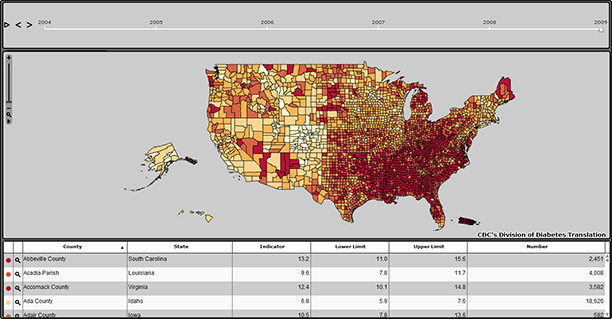
Screenshot of the default display of US county data on diabetes and its risk factors in the Diabetes Interactive Atlas (www.cdc.gov/diabetes/atlas/countydata/atlas.html).

The atlas for county data (www.cdc.gov/diabetes/atlas/countydata/atlas.html) displays a map of the United States showing crude and age-adjusted estimates of the prevalence and incidence of diabetes and the prevalence of obesity and physical inactivity by county. It also presents data on the prevalence of diabetes, obesity, and physical inactivity by sex. In this atlas, the user can interact with maps and data tables. The user can select an indicator to be displayed in both the map and the table by clicking on the “Indicator” button and selecting from the drop-down list. The default display shows all US counties (Figure 1). To display county data by state, the user would click on the “Select State” button to select a state from the drop-down list of all states. 

The data table can be sorted according to any column heading in the table, including county name, state name, indicator value, lower and upper confidence limits of indicator value, and total number of adults by indicator. Row or multiple rows selected on the data table will be highlighted on the map. Likewise, if the user clicks on a county or multiple counties or rolls over a county in the map, those counties are highlighted in the table. The “Legend Settings” button allows the user to choose different data classifications (ie, equal intervals, continuous, natural breaks, or quantiles) and different numbers of data classes (from 2 to 10 classes) to view an indicator. The time animation bar located near the top of the Web page allows the user to view trends over time for the United States and to select any 1 year for viewing. Other features of the atlas are functions for zooming in and out, printing, exporting, and downloading, and a tutorial, “How to Use the Atlas.”

### County rankings

The atlas for county rankings (www.cdc.gov/diabetes/atlas/countyrank/atlas.html) has all of the features of the atlas for county data. It shows a map of the United States by county and indicates whether the age-adjusted rates (of the chosen indicator) in the counties rank above or below or are no different from the US median rates. Ranks for county data on diagnosed diabetes, obesity, and physical inactivity are available; however, ranks are not available for county estimates of diabetes prevalence by sex or for incidence because most of these measures have a coefficient of variation greater than 0.3. The rank estimates have large confidence intervals and are highly variable (20). These confidence intervals need to be considered before reaching conclusions about counties based on ranks. For example, in 2010 Cook County, Illinois, ranked 1,508th in the prevalence of diagnosed diabetes. However, the lower and upper limits of the rank for Cook County were 1,224th (5th percentile) and 1,774st (95th percentile).

### Maps and motion charts

#### Maps and motion charts: the “All States” option

**Figure 2:**
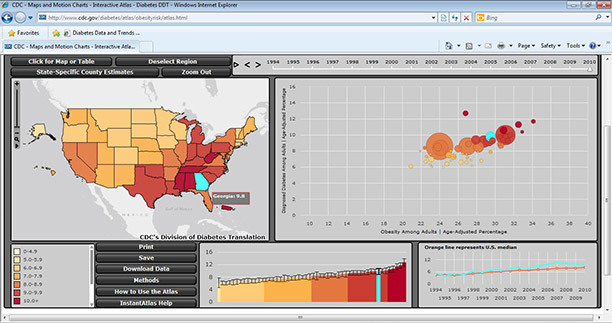
Screenshot of the default display of the maps and motion charts on diabetes and its risk factors for all states in the Diabetes Interactive Atlas (www.cdc.gov/diabetes/atlas/obesityrisk/atlas.html).

The Web page “Maps and Motion Charts — All States” (www.cdc.gov/diabetes/atlas/obesityrisk/atlas.html) presents more information and the default display is more complicated than other displays of the atlas. The default display (Figure 2) shows 4 images depicting data on all 50 states: 1) a choropleth map of age-adjusted diabetes prevalence (which can be switched to a table view), 2) a bubble chart of age-adjusted diabetes and obesity prevalence, 3) a bar chart of age-adjusted diabetes prevalence, and 4) a chart of the US median age-adjusted prevalence of diabetes from 1994 to 2010. In addition, the page shows a time animation bar. The default display of the state data is the national view; however, the user can click on the “Select Region” button and view state data by US census region or division. The bar chart shows the indicator value with lower and upper confidence limits for each state for each year. The degree of uncertainty for each estimate is discerned by examining the error bars, which indicate lower and upper limits. For example, a precise estimate will have a narrow interval. The time-series chart displays an orange trend line that represents the US median prevalence for each year. When the user moves the mouse over a state in the map, bubble chart, or bar chart, the trend line for that state is shown and can be compared with the US median.

By clicking the “Play” button on the time animation bar, the user can see changes in an indicator over time and across states in the map, bubble chart, and bar chart. The motion of the bubble chart allows investigation of the complex interplay of data between an indicator and a known risk factor, obesity. The parameters defined for the default motion chart data are the following: the *x*-axis is the age-adjusted percentage of obesity, the *y*-axis is the age-adjusted prevalence of diabetes, the bubble color indicates data class, and the bubble size is proportional to the number of adults with diabetes. Although the *x*-axis always indicates obesity, the user can select a different indicator (ie, diabetes incidence or physical inactivity) for the *y*-axis.

The user can select multiple states by holding down the “Control” key while clicking the mouse. Those states will be highlighted in all the data frames. Other features in the atlas include zooming in and out, printing, exporting, downloading, and an online tutorial.

#### Maps and motion charts: the “Select a State” option

By clicking on “Select a State” (www.cdc.gov/diabetes/atlas/obesityrisk/county_statelist.html), the user is taken to a page that displays the names of the 50 states, and by clicking on a state, the user is taken to county-level age-adjusted estimates for the selected state. The “Select a State” Web pages have all of the components and functionality found in “All States.” The atlas includes a transparent map tool that helps users who may know a city name but not a county name. By moving the tool’s slider bar to high transparency, the user can find the city on the background map, and then click on the city to highlight the county and county name.

## Summary

The goals for the Diabetes Interactive Atlas were to improve visualization of geographic patterns, improve observation of trends, and demonstrate concomitant geographic and temporal growth of diabetes and obesity. To this end, the Diabetes Interactive Atlas has tools to make navigation and data investigation easy and engaging. We considered several design issues in the development of the atlas to ensure ease of navigation and data interpretation. The design considerations outlined in this article helped in creating an atlas application that is a useful tool for health professionals in interpreting trends and in downloading maps and graphs for presentations and reports. Health professionals will be able to identify at-risk populations by using various graphical displays to assist them in designing and targeting public health policies and interventions.

Feedback from online surveys used by the NDSS and e-mailed queries and comments will help us to understand how best to make online information more easily obtained and useful. On the basis of this feedback, we can make modifications to the atlas that will help users to interpret and use the data in an effort to better understand the burden of diabetes and related risk factors.
